# *trans*-Bis(*N*′-isopropyl­idene­benzo­hydrazidato-κ^2^ *N*′,*O*)bis­(pyridine-κ*N*)nickel(II)

**DOI:** 10.1107/S1600536811024214

**Published:** 2011-06-25

**Authors:** Chang-Zheng Zheng, Liang Wang, Juan Liu

**Affiliations:** a College of Environment and Chemical Engineering, Xi’an Polytechnic University, 710048 Xi’an, Shaanxi, People’s Republic of China

## Abstract

The complex mol­ecule of the title compound, [Ni(C_10_H_11_N_2_O)_2_(C_5_H_5_N)_2_], has a crystallographically imposed centre of symmetry. The Ni^II^ atom is coordinated in a distorted octa­hedral geometry by the O and N atoms of two *trans* arranged anionic bidentate hydrazone ligands forming the equatorial plane and by the N atoms of two pyridine mol­ecules at the axial positions. In the crystal, inter­molecular C—H⋯N hydrogen bonds link the mol­ecules into columns parallel to the *b* axis.

## Related literature

For the biological and coordination properties of aroylhydrazones, see: Ali *et al.* (2004 ▶); Carcelli *et al.* (1995 ▶); Cheng *et al.* (1996 ▶); Zhang *et al.* (2011 ▶).
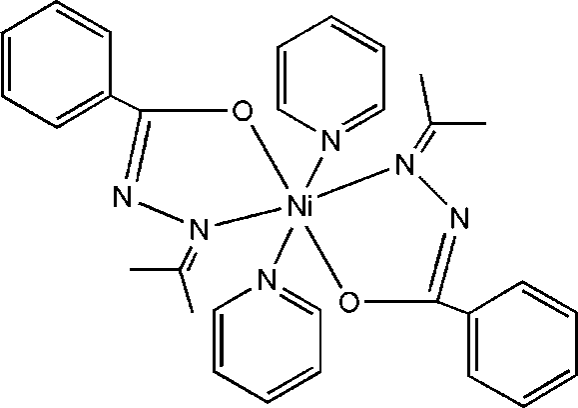


## Experimental

### 

#### Crystal data

[Ni(C_10_H_11_N_2_O)_2_(C_5_H_5_N)_2_]
*M* *_r_* = 567.33
Monoclinic, 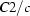

*a* = 15.419 (5) Å
*b* = 9.242 (3) Å
*c* = 21.295 (9) Å
β = 109.924 (5)°
*V* = 2853.1 (18) Å^3^
*Z* = 4
Mo *K*α radiation
μ = 0.72 mm^−1^
*T* = 298 K
0.23 × 0.14 × 0.12 mm

#### Data collection

Bruker SMART CCD area-detector diffractometer
Absorption correction: multi-scan (*SADABS*; Sheldrick, 1996 ▶) *T*_min_ = 0.852, *T*_max_ = 0.919
7093 measured reflections
2514 independent reflections
2285 reflections with *I* > 2σ(*I*)
*R*_int_ = 0.019

#### Refinement

*R*[*F* ^2^ > 2σ(*F* ^2^)] = 0.028
*wR*(*F* ^2^) = 0.078
*S* = 1.10
2514 reflections
178 parameters
H-atom parameters constrained
Δρ_max_ = 0.24 e Å^−3^
Δρ_min_ = −0.32 e Å^−3^

### 

Data collection: *SMART* (Bruker, 1996 ▶); cell refinement: *SAINT* (Bruker, 1996 ▶); data reduction: *SAINT*; program(s) used to solve structure: *SHELXS97* (Sheldrick, 2008 ▶); program(s) used to refine structure: *SHELXL97* (Sheldrick, 2008 ▶); molecular graphics: *SHELXTL* (Sheldrick, 2008 ▶); software used to prepare material for publication: *SHELXTL*.

## Supplementary Material

Crystal structure: contains datablock(s) I, global. DOI: 10.1107/S1600536811024214/rz2613sup1.cif

Structure factors: contains datablock(s) I. DOI: 10.1107/S1600536811024214/rz2613Isup2.hkl

Additional supplementary materials:  crystallographic information; 3D view; checkCIF report

## Figures and Tables

**Table 1:** Hydrogen-bond geometry (Å, °)

*D*—H⋯*A*	*D*—H	H⋯*A*	*D*⋯*A*	*D*—H⋯*A*
C13—H13⋯N1^i^	0.93	2.50	3.382 (3)	157

Symmetry code: (i) 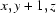.

**Fig. 1.**
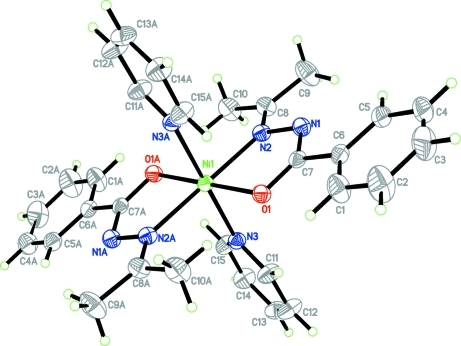
The molecular structure of the title compound, showing displacement ellipsoids drawn at the 30% probability level. H atoms are presented as small spheres of arbitrary radius. Symmetry code: (A) -x, 1-y, -z.

## supplementary crystallographic information

### Comment

Hydrazones are an important class of Schiff bases compounds which has attracted much attention because of their biological activities (Carcelli *et al.*, 1995), chemical and industrial versatility, and strong tendency to chelate to transition metals (Zhang *et al.*, 2011; Ali *et al.*, 2004; Cheng *et al.*, 1996).  As an extension of our work on the structural characterization of aroylhydrazone derivatives, the title compound was synthesized and its crystal structure is reported here.

In the title compound, the complex molecule has crystallographically imposed centre of symmetry (Fig. 1). The coordination polyhedron about the nickel metal is distorted octahedral, with the N, O atoms of two *trans*-arranged anionic bidentate hydrazone ligands at the equatorial plane and by the N atoms of two pyridine molecules occupying the axial positions. In the crystal structure, complex molecules are linked by intermolecular C—H···N hydrogen bonds (Table 1) into columns parallel to the *b* axis.

### Experimental

Ethyl benzoate (6.00 g, 0.04 mol) was dissolved in ethnol (30 ml) at room temperature and heated at 363 K, followed by the addition of hydrazine hydrate (2.40 g, 0.048 mol). Subsequently, the mixture was refluxed for 9 h, and then cooled to room temperature. The crystals precipitated were collected by filtration. The product was recrystallized from ethanol and dried under reduced pressure to give benzoylhydrazine. Benzoylhydrazine (3.40 g, 0.025 mol) was dissolved in ethanol (20 ml) at room temperature and heated at 363 K, followed by the addition of dimethyl ketone (1.45 g, 0.025 mol). Subsequently, the mixture was refluxed for 10 h, and then cooled to room temperature. The solid phase precipitated was collected by filtration. The product was recrystallized from ethanol and dried under reduced pressure to give *N*'-[(*E*)- dimethylketone]-benzohydrazide. A mixture of *N*'-[(*E*)-dimethylketone]-benzohydrazide (0.018 g, 0.10 mmol), NiC_l2_.6H_2_O (0.024 g, 0.10 mmol), pyridine (0.0079 g, 0.10 mmol), H_2_O (5.00 ml) and several drop of methanol was placed in a Parr Teflon-lined stainless steel vessel (25 ml), and then the vessel was sealed and heated at 393 K for 3 d. After the mixture was slowly cooled to room temperature, red crystals suitable for X-ray analysis were obtained (yield 37%).

### Refinement

All H atoms were positioned geometrically and treated as riding on their parent atoms,with C—H = 0.93-0.96 Å, and with *U*_iso_(H) = 1.2*U*_eq_(C) or 1.5*U*_eq_(C) for methyl H atoms.

### Crystal data

**Table:**

[Ni(C_10_H_11_N_2_O)_2_(C_5_H_5_N)_2_]	*F*(000) = 1192
*M**_r_* = 567.33	*D*_x_ = 1.321 Mg m^−^^3^
Monoclinic, *C*2/*c*	Mo *K*α radiation, λ = 0.71073 Å
Hall symbol: -C 2yc	Cell parameters from 5423 reflections
*a* = 15.419 (5) Å	θ = 2.6–28.5°
*b* = 9.242 (3) Å	µ = 0.72 mm^−^^1^
*c* = 21.295 (9) Å	*T* = 298 K
β = 109.924 (5)°	Block, red
*V* = 2853.1 (18) Å^3^	0.23 × 0.14 × 0.12 mm
*Z* = 4	

### Data collection

**Table:**

Bruker SMART CCD area-detector diffractometer	2514 independent reflections
Radiation source: fine-focus sealed tube	2285 reflections with *I* > 2σ(*I*)
graphite	*R*_int_ = 0.019
φ and ω scans	θ_max_ = 25.1°, θ_min_ = 2.0°
Absorption correction: multi-scan (*SADABS*; Sheldrick, 1996)	*h* = −18→16
*T*_min_ = 0.852, *T*_max_ = 0.919	*k* = −10→8
7093 measured reflections	*l* = −24→25

### Refinement

**Table:**

Refinement on *F*^2^	Secondary atom site location: difference Fourier map
Least-squares matrix: full	Hydrogen site location: inferred from neighbouring sites
*R*[*F*^2^ > 2σ(*F*^2^)] = 0.028	H-atom parameters constrained
*wR*(*F*^2^) = 0.078	*w* = 1/[σ^2^(*F*_o_^2^) + (0.0334*P*)^2^ + 2.5175*P*] where *P* = (*F*_o_^2^ + 2*F*_c_^2^)/3
*S* = 1.10	(Δ/σ)_max_ < 0.001
2514 reflections	Δρ_max_ = 0.24 e Å^−^^3^
178 parameters	Δρ_min_ = −0.32 e Å^−^^3^
0 restraints	Extinction correction: *SHELXL97* (Sheldrick, 2008)
Primary atom site location: structure-invariant direct methods	Extinction coefficient: 0.0113 (15)

### Special details

**Table:**

Geometry. All e.s.d.'s (except the e.s.d. in the dihedral angle between two l.s. planes) are estimated using the full covariance matrix. The cell e.s.d.'s are taken into account individually in the estimation of e.s.d.'s in distances, angles and torsion angles; correlations between e.s.d.'s in cell parameters are only used when they are defined by crystal symmetry. An approximate (isotropic) treatment of cell e.s.d.'s is used for estimating e.s.d.'s involving l.s. planes.
Refinement. Refinement of *F*^2^ against ALL reflections. The weighted *R*-factor *wR* and goodness of fit *S* are based on *F*^2^, conventional *R*-factors *R* are based on *F*, with *F* set to zero for negative *F*^2^. The threshold expression of *F*^2^ > σ(*F*^2^) is used only for calculating *R*-factors(gt) *etc*. and is not relevant to the choice of reflections for refinement. *R*-factors based on *F*^2^ are statistically about twice as large as those based on *F*, and *R*- factors based on ALL data will be even larger.

### Fractional atomic coordinates and isotropic or equivalent isotropic displacement parameters (Å^2^)

**Table:**

	*x*	*y*	*z*	*U*_iso_*/*U*_eq_	
Ni1	0.0000	0.5000	0.0000	0.03337 (12)	
O1	0.12440 (8)	0.44811 (14)	0.06671 (6)	0.0385 (3)	
N1	0.04126 (10)	0.34430 (16)	0.12653 (8)	0.0393 (4)	
N2	−0.03583 (10)	0.40836 (16)	0.07882 (7)	0.0375 (3)	
N3	0.00804 (10)	0.71169 (16)	0.04278 (7)	0.0383 (3)	
C1	0.28411 (15)	0.3172 (3)	0.14883 (12)	0.0636 (6)	
H1	0.2865	0.3759	0.1140	0.076*	
C2	0.36373 (18)	0.2488 (4)	0.18906 (15)	0.0871 (9)	
H2	0.4190	0.2619	0.1812	0.104*	
C3	0.36080 (19)	0.1624 (3)	0.23998 (14)	0.0788 (8)	
H3	0.4138	0.1154	0.2668	0.095*	
C4	0.27956 (18)	0.1450 (3)	0.25151 (12)	0.0665 (7)	
H4	0.2775	0.0861	0.2864	0.080*	
C5	0.20085 (15)	0.2138 (2)	0.21199 (10)	0.0504 (5)	
H5	0.1462	0.2020	0.2208	0.061*	
C6	0.20201 (13)	0.2999 (2)	0.15964 (9)	0.0397 (4)	
C7	0.11672 (12)	0.37097 (18)	0.11420 (9)	0.0348 (4)	
C8	−0.11104 (14)	0.3927 (2)	0.09097 (10)	0.0474 (5)	
C9	−0.11699 (18)	0.3144 (3)	0.15068 (13)	0.0741 (8)	
H9A	−0.1454	0.2217	0.1373	0.111*	
H9B	−0.1534	0.3699	0.1707	0.111*	
H9C	−0.0561	0.3012	0.1825	0.111*	
C10	−0.19837 (15)	0.4545 (3)	0.04456 (13)	0.0654 (6)	
H10A	−0.1871	0.4978	0.0071	0.098*	
H10B	−0.2210	0.5267	0.0675	0.098*	
H10C	−0.2434	0.3790	0.0291	0.098*	
C11	0.08853 (14)	0.7625 (2)	0.08221 (11)	0.0529 (5)	
H11	0.1406	0.7046	0.0902	0.063*	
C12	0.09888 (16)	0.8958 (2)	0.11177 (13)	0.0646 (6)	
H12	0.1566	0.9265	0.1397	0.078*	
C13	0.02369 (17)	0.9830 (2)	0.09987 (12)	0.0551 (6)	
H13	0.0289	1.0742	0.1193	0.066*	
C14	−0.05893 (15)	0.9331 (2)	0.05897 (12)	0.0554 (5)	
H14	−0.1115	0.9902	0.0496	0.066*	
C15	−0.06440 (14)	0.7977 (2)	0.03149 (11)	0.0497 (5)	
H15	−0.1216	0.7648	0.0037	0.060*	

### Atomic displacement parameters (Å^2^)

**Table:**

	*U*^11^	*U*^22^	*U*^33^	*U*^12^	*U*^13^	*U*^23^
Ni1	0.02844 (18)	0.03087 (18)	0.03696 (19)	0.00162 (12)	0.00618 (13)	0.00258 (13)
O1	0.0330 (6)	0.0372 (7)	0.0409 (7)	0.0014 (5)	0.0068 (5)	0.0065 (6)
N1	0.0378 (8)	0.0370 (8)	0.0406 (8)	0.0051 (7)	0.0101 (7)	0.0043 (7)
N2	0.0345 (8)	0.0331 (8)	0.0427 (8)	0.0019 (6)	0.0106 (7)	0.0009 (6)
N3	0.0373 (8)	0.0346 (8)	0.0404 (8)	−0.0003 (7)	0.0099 (7)	0.0009 (6)
C1	0.0458 (12)	0.0827 (17)	0.0591 (14)	0.0196 (12)	0.0138 (10)	0.0214 (12)
C2	0.0473 (14)	0.126 (3)	0.0819 (19)	0.0321 (16)	0.0147 (13)	0.0277 (19)
C3	0.0627 (16)	0.0872 (19)	0.0689 (17)	0.0342 (14)	−0.0003 (13)	0.0178 (14)
C4	0.0732 (17)	0.0562 (14)	0.0525 (13)	0.0089 (12)	−0.0014 (12)	0.0165 (11)
C5	0.0527 (12)	0.0444 (11)	0.0458 (11)	0.0008 (10)	0.0061 (9)	0.0033 (9)
C6	0.0409 (10)	0.0333 (9)	0.0375 (10)	0.0046 (8)	0.0039 (8)	−0.0028 (7)
C7	0.0371 (9)	0.0268 (9)	0.0354 (9)	0.0020 (7)	0.0059 (7)	−0.0035 (7)
C8	0.0410 (10)	0.0492 (11)	0.0549 (12)	0.0033 (9)	0.0201 (9)	0.0051 (9)
C9	0.0614 (15)	0.094 (2)	0.0782 (17)	0.0140 (14)	0.0386 (13)	0.0308 (15)
C10	0.0394 (11)	0.0851 (17)	0.0734 (16)	0.0077 (12)	0.0213 (11)	0.0201 (14)
C11	0.0404 (11)	0.0412 (11)	0.0693 (14)	0.0015 (9)	0.0087 (10)	−0.0077 (10)
C12	0.0497 (13)	0.0499 (13)	0.0834 (17)	−0.0085 (10)	0.0086 (12)	−0.0191 (12)
C13	0.0625 (14)	0.0358 (11)	0.0698 (15)	−0.0047 (10)	0.0261 (12)	−0.0104 (10)
C14	0.0507 (12)	0.0463 (12)	0.0691 (14)	0.0106 (10)	0.0204 (11)	−0.0057 (11)
C15	0.0387 (10)	0.0480 (11)	0.0577 (12)	0.0028 (9)	0.0104 (9)	−0.0089 (10)

### Geometric parameters (Å, °)

**Table:**

Ni1—O1^i^	2.0175 (13)	C5—C6	1.374 (3)
Ni1—O1	2.0175 (13)	C5—H5	0.9300
Ni1—N2	2.1148 (16)	C6—C7	1.493 (2)
Ni1—N2^i^	2.1148 (16)	C8—C10	1.486 (3)
Ni1—N3	2.1439 (16)	C8—C9	1.493 (3)
Ni1—N3^i^	2.1439 (16)	C9—H9A	0.9600
O1—C7	1.275 (2)	C9—H9B	0.9600
N1—C7	1.301 (2)	C9—H9C	0.9600
N1—N2	1.404 (2)	C10—H10A	0.9600
N2—C8	1.279 (2)	C10—H10B	0.9600
N3—C15	1.325 (2)	C10—H10C	0.9600
N3—C11	1.325 (2)	C11—C12	1.368 (3)
C1—C6	1.371 (3)	C11—H11	0.9300
C1—C2	1.387 (3)	C12—C13	1.363 (3)
C1—H1	0.9300	C12—H12	0.9300
C2—C3	1.360 (4)	C13—C14	1.356 (3)
C2—H2	0.9300	C13—H13	0.9300
C3—C4	1.366 (4)	C14—C15	1.372 (3)
C3—H3	0.9300	C14—H14	0.9300
C4—C5	1.375 (3)	C15—H15	0.9300
C4—H4	0.9300		
			
O1^i^—Ni1—O1	180.00 (9)	C4—C5—H5	119.6
O1^i^—Ni1—N2	102.19 (6)	C1—C6—C5	118.16 (19)
O1—Ni1—N2	77.81 (6)	C1—C6—C7	119.95 (18)
O1^i^—Ni1—N2^i^	77.81 (6)	C5—C6—C7	121.87 (18)
O1—Ni1—N2^i^	102.19 (6)	O1—C7—N1	126.67 (16)
N2—Ni1—N2^i^	180.00 (8)	O1—C7—C6	117.41 (16)
O1^i^—Ni1—N3	89.18 (6)	N1—C7—C6	115.89 (16)
O1—Ni1—N3	90.82 (6)	N2—C8—C10	120.02 (18)
N2—Ni1—N3	91.21 (6)	N2—C8—C9	123.37 (19)
N2^i^—Ni1—N3	88.79 (6)	C10—C8—C9	116.61 (18)
O1^i^—Ni1—N3^i^	90.82 (6)	C8—C9—H9A	109.5
O1—Ni1—N3^i^	89.18 (6)	C8—C9—H9B	109.5
N2—Ni1—N3^i^	88.79 (6)	H9A—C9—H9B	109.5
N2^i^—Ni1—N3^i^	91.21 (6)	C8—C9—H9C	109.5
N3—Ni1—N3^i^	180.00 (8)	H9A—C9—H9C	109.5
C7—O1—Ni1	111.61 (11)	H9B—C9—H9C	109.5
C7—N1—N2	111.65 (14)	C8—C10—H10A	109.5
C8—N2—N1	114.09 (16)	C8—C10—H10B	109.5
C8—N2—Ni1	134.88 (14)	H10A—C10—H10B	109.5
N1—N2—Ni1	110.93 (11)	C8—C10—H10C	109.5
C15—N3—C11	116.89 (17)	H10A—C10—H10C	109.5
C15—N3—Ni1	123.21 (13)	H10B—C10—H10C	109.5
C11—N3—Ni1	119.90 (13)	N3—C11—C12	123.2 (2)
C6—C1—C2	121.1 (2)	N3—C11—H11	118.4
C6—C1—H1	119.5	C12—C11—H11	118.4
C2—C1—H1	119.5	C13—C12—C11	119.3 (2)
C3—C2—C1	119.8 (3)	C13—C12—H12	120.4
C3—C2—H2	120.1	C11—C12—H12	120.4
C1—C2—H2	120.1	C14—C13—C12	118.2 (2)
C2—C3—C4	119.6 (2)	C14—C13—H13	120.9
C2—C3—H3	120.2	C12—C13—H13	120.9
C4—C3—H3	120.2	C13—C14—C15	119.5 (2)
C3—C4—C5	120.5 (2)	C13—C14—H14	120.3
C3—C4—H4	119.7	C15—C14—H14	120.3
C5—C4—H4	119.7	N3—C15—C14	123.00 (19)
C6—C5—C4	120.8 (2)	N3—C15—H15	118.5
C6—C5—H5	119.6	C14—C15—H15	118.5
			
N2—Ni1—O1—C7	9.82 (11)	C3—C4—C5—C6	0.8 (4)
N2^i^—Ni1—O1—C7	−170.18 (11)	C2—C1—C6—C5	0.7 (4)
N3—Ni1—O1—C7	100.88 (12)	C2—C1—C6—C7	−178.0 (2)
N3^i^—Ni1—O1—C7	−79.12 (12)	C4—C5—C6—C1	−1.2 (3)
C7—N1—N2—C8	−175.97 (16)	C4—C5—C6—C7	177.42 (19)
C7—N1—N2—Ni1	7.01 (17)	Ni1—O1—C7—N1	−10.2 (2)
O1^i^—Ni1—N2—C8	−5.3 (2)	Ni1—O1—C7—C6	168.00 (12)
O1—Ni1—N2—C8	174.7 (2)	N2—N1—C7—O1	1.9 (2)
N3—Ni1—N2—C8	84.12 (19)	N2—N1—C7—C6	−176.30 (14)
N3^i^—Ni1—N2—C8	−95.88 (19)	C1—C6—C7—O1	−0.9 (3)
O1^i^—Ni1—N2—N1	170.85 (10)	C5—C6—C7—O1	−179.55 (17)
O1—Ni1—N2—N1	−9.15 (10)	C1—C6—C7—N1	177.46 (19)
N3—Ni1—N2—N1	−99.72 (11)	C5—C6—C7—N1	−1.2 (3)
N3^i^—Ni1—N2—N1	80.28 (11)	N1—N2—C8—C10	−179.78 (19)
O1^i^—Ni1—N3—C15	16.25 (16)	Ni1—N2—C8—C10	−3.7 (3)
O1—Ni1—N3—C15	−163.75 (16)	N1—N2—C8—C9	0.4 (3)
N2—Ni1—N3—C15	−85.93 (16)	Ni1—N2—C8—C9	176.48 (18)
N2^i^—Ni1—N3—C15	94.07 (16)	C15—N3—C11—C12	1.2 (3)
O1^i^—Ni1—N3—C11	−163.18 (16)	Ni1—N3—C11—C12	−179.37 (19)
O1—Ni1—N3—C11	16.82 (16)	N3—C11—C12—C13	−1.0 (4)
N2—Ni1—N3—C11	94.64 (16)	C11—C12—C13—C14	0.2 (4)
N2^i^—Ni1—N3—C11	−85.36 (16)	C12—C13—C14—C15	0.4 (4)
C6—C1—C2—C3	0.3 (5)	C11—N3—C15—C14	−0.5 (3)
C1—C2—C3—C4	−0.7 (5)	Ni1—N3—C15—C14	−179.99 (17)
C2—C3—C4—C5	0.1 (4)	C13—C14—C15—N3	−0.2 (4)

Symmetry codes: (i) −*x*, −*y*+1, −*z*.

### Hydrogen-bond geometry (Å, °)

**Table:**

*D*—H···*A*	*D*—H	H···*A*	*D*···*A*	*D*—H···*A*
C13—H13···N1^ii^	0.93	2.50	3.382 (3)	157

Symmetry codes: (ii) *x*, *y*+1, *z*.
